# Parallel adaptation prompted core-periphery divergence of *Ammopiptanthus mongolicus*

**DOI:** 10.3389/fpls.2022.956374

**Published:** 2022-08-24

**Authors:** Yong-Zhi Yang, Min-Xin Luo, Li-Dong Pang, Run-Hong Gao, Jui-Tse Chang, Pei-Chun Liao

**Affiliations:** ^1^College of Forestry, Inner Mongolia Agricultural University, Huhhot, China; ^2^School of Life Sciences, National Taiwan Normal University, Taipei, Taiwan; ^3^College Resource and Environmental Economics, Inner Mongolia University of Finance and Economics, Huhhot, China

**Keywords:** adaptive optima shift, core-periphery hypothesis, edge effect, genome-environment association, local adaptation, range expansion

## Abstract

Range expansion requires peripheral populations to shift adaptive optima to breach range boundaries. Opportunities for range expansion can be assessed by investigating the associations of core-periphery environmental and genetic differences. This study investigates differences in the core-periphery adaptation of *Ammopiptanthus mongolicus*, a broad-leaved evergreen shrub species in a relatively homogeneous temperate Asian desert environment, to explore the environmental factors that limit the expansion of desert plants. Temperate deserts are characterized by severe drought, a large diurnal temperature range, and seasonality. Long-standing adaptation to the harsh desert environment may confine the genetic diversity of *A. mongolicus*, despite its distribution over a wide range of longitude, latitude, and altitude. Since range edges defined by climate niches may have different genetic responses to environmental extremes, we compared genome-wide polymorphisms between nine environmental core populations and ten fragmented peripheral populations to determine the “adaptive peripheral” populations. At least four adaptive peripheral populations had similar genetic-environmental association patterns. High elevations, summer drought, and winter cold were the three main determinants of converging these four adaptive peripheral populations. Elevation mainly caused similar local climates among different geographic regions. Altitudinal adaptation resulting from integrated environmental-genetic responses was a breakthrough in breaching niche boundaries. These peripheral populations are also located in relatively humid and warmer environments. Relaxation of the drought and cold constraints facilitated the genetic divergence of these peripheral populations from the core population’s adaptive legacy. We conclude that pleiotropic selection synchronized adaptative divergence to cold and drought vs. warm and humid environments between the core and peripheral populations. Such parallel adaptation of peripheral populations relies on selection under a background of abundant new variants derived from the core population’s standing genetic variation, i.e., integration of genetic surfing and local adaptation.

## Introduction

Different responses of populations to environmental stress may result in adaptive divergence of species (Bridle and Vines, 2007). Many evolutionary biologists believe this process could be a primary stage of speciation. Peripheral populations live on the edge of the optimal range and are usually under different selection pressures than core populations (Duncan et al., 2015), making it a marvellous model system for studying adaptive divergence (Eckert et al., 2008). Ecological tolerance sustains the survival of marginal populations at low growth rates and will be negative outside the range boundary (Eckert et al., 2008). Only adaptive optima shift at the expanding range front, especially when the environment fluctuates (Whitlock, 1997), will have the opportunity to break through the boundaries (Burton and Travis, 2008).

Ecological niche modelling (ENM) theoretically predicts a species’ distribution range under the assumption of phylogenetic niche conservatism (PNC), i.e., slow temporal changes in the hypothetical fundamental niche (Soberón and Nakamura, 2009). In practice, however, the range margins of extreme environments to which a species adapts might violate the PNC and diverge from the core niches, resulting in phylogenetic niche divergence (PND). Populations at these PND margins may have higher adaptability to be founders for range expansion (Dudaniec et al., 2018; Stuart et al., 2021), signifying that signatures of adaptive divergence can be expected in genomes of PND populations.

Environmental heterogeneity may lead to fundamental niche differences in organisms with overlapping geographic space (G-space) (Brown and Carnaval, 2019). Thus, environmental space (E-space) is recommended to define the distribution ranges of adaptive peaks (Brown and Carnaval, 2019). The E-space concept regards peripheral (marginal) populations as living in suboptimal or even poor habitats, where the harsher environment may select the margins, diverging them from the core population and resulting in optimal shifts in adaptive peaks (Macdonald et al., 2017). This process could be facilitated by strong drift with intermediate gene flow in small peripheral populations (Alleaume-Benharira et al., 2006; Garant et al., 2007; Polechová, 2022) and increased frequency of new mutations by genetic surfing (Excoffier and Ray, 2008). If environmental selection pressures outweigh adaptive responses, the genetic load of peripheral populations will increase. By contrast, if the timely genetic change occurs through genetic surfing and local adaptation, the peripheral population will have the opportunity to become the new fitness peak (Hoffmann and Blows, 1994). Accordingly, we can predict higher core-periphery divergence in the genome.

The desert environment, characterized by water deficits and large temperature differences, restricts plant growth for long periods (James et al., 2005). Under such prolonged adversity, the core population of a species must either grow in profitable regions (such as oases and riparian areas) or have long adapted to the adversity (Bechtold, 2018; Kirschner et al., 2021). The former represents spatial selection (under PNC), while the latter requires genetic change (i.e., PND). Under PNC, spatial selection reduces genetic variation in peripheral populations without adaptive divergence from the core (Price et al., 2011); by contrast, adaptive changes lead peripheral populations to new adaptive peaks under PND (Grossenbacher et al., 2014; Friis et al., 2018). In this study, we compared the genetic diversity and compositions of core and peripheral populations of the desert plant *Ammopiptanthus mongolicus* (Maxim. ex Kom.) S. H. Cheng (Leguminosae), a second-grade vulnerable (VU) plant of the China Red List of Threatened Species (the Red Book) (Fu, 1992), to assess whether the peripheral populations have adaptively diverged to break through the space limitation for range expansion.

Under PNC, the distribution range of *A. mongolicus* tends to expand under climate warming and is affected mainly by temperature seasonality and precipitation in the winter (coldest quarter) and summer (wettest month) (Du et al., 2021). Since precipitation varies with longitude in inland Asia, Du et al. (2021) predicted different expansion routes of *A. mongolicus* under different warming scenarios: eastward under the mild warming scenario (greenhouse gas emission scenario RCP4.5) but westward under severe warming (RCP8.5) (Du et al., 2021). Its heterocarpy (dehiscent-flat, dehiscent-twisted, and indehiscent-flat diaspores) ensures and diversifies propagation in deserts (Yang et al., 2021). In addition to wind speed and ground substrate, which may affect spread distance (Yang et al., 2021), the geographic distance (Ge et al., 2005), and local environments (e.g., soil organic matter, total nitrogen, and summer rainfall) (Liu et al., 2017; Jiang et al., 2019) also determine its spatial-genetic structure, i.e., isolation-by-distance (IBD) and isolation-by-environment (IBE). As the only broad-leaved perennial evergreen shrub in eastern Central Asian temperate deserts, *A. mongolicus* must be able to endure harsh winters. Several genetic and physiological studies have shown excellent cold (and drought) resistance gene expression in *A. mongolicus* (Liu et al., 2013b; Wu et al., 2014; Pang et al., 2019), suggesting that this species may have undergone not only spatial selection but also genetic change for local adaptation.

Genome-environment associations (GEAs), also known as genotype-environment associations, provide an opportunity to quantify the core-periphery adaptive divergence of *A. mongolicus*. Briefly, GEA analysis is a non-hypothesis-driven genome-wide association approach to determining selective drivers by identifying genes [or even anonymous single nucleotide polymorphisms (SNPs)] correlated with individual environmental predictors (Rellstab et al., 2015). The selection strength is quantified based on the significance of the correlation and the number of correlated genes (SNPs). Thus, this strategy can be used to predict the environmental factors driving genetic divergence in non-model species. This study adopted both univariate (latent factor mixed model, LFMM) and multivariate regressions (redundancy analysis, RDA) for GEA analysis. The former corrects for the underlying population structure (Frichot et al., 2013), and the latter overcomes small environmental differences among populations (Forester et al., 2018). These two methods are appropriate for the sharp genetic structure of *A. mongolicus* (Ge et al., 2005; Jiang et al., 2019) and the relatively homogeneous desert environment.

We hypothesized that genetic surfing in peripheral populations facilitates adaptive divergence from the core populations. Despite the high dispersibility of *A. mongolicus*, environmental heterogeneity has been proposed as a limiting factor in its dispersal. New variants arising from genetic surfing may drive the expansion and colonization of peripheral populations. We adopted the E-space concept to define the core and peripheral populations and compare their genomic diversity patterns. Selective drivers that diverged the core and peripheral populations were then determined using the GEA strategy.

## Materials and methods

### Ecological niche modelling and climate niche determination

We reconstructed the potential distribution according to field investigations, specimen records, Jiang et al. (2019) and Chai et al. (2021) (Supplementary Table 1). Although Du et al. (2021) published ENM of *A. mongolicus*, they did not provide the exact location of the modelling. We used a topographic variable, the altitude (m), and 55 environmental variables at a spatial resolution of 2.5 arc-minutes (approximately 4.5 km × 4.5 km at the equator) from WorldClim ver. 2 (Fick and Hijmans, 2017) for ENM, including 19 bioclimatic variables, 12 monthly solar radiations (kJ m^–2^ day^–1^), 12 monthly water vapour pressures (kPa), and 12 monthly wind speeds (m s^–1^). Variance inflation factor (VIF) analysis was first conducted to remove collinear factors with VIF < 6, which left seven factors: altitude (alt), March solar radiation (srad03), January water vapour pressure (vapr01), precipitation in the wettest month (bio13), mean diurnal range (bio2), mean temperature in the driest quarter (bio9), and precipitation in the driest month (bio14). We conducted the maximum entropy model in MaxEnt v3.3.3 (Phillips and Dudík, 2008) and the R package raster (Hijmans and van Etten, 2014). To reduce sampling bias, we thinned the occurrence data using the R package spThin (Aiello-Lammens et al., 2014) to ensure that the records were separated by at least 5 km. A maximum of 1,000 iterations of each prediction were conducted. We randomly chose 25% of the species occurrence data as testing samples. One regularization multiplier, 10,000 background points, and the default auto feature were used to create models for each set of predictions. Maximum training sensitivity plus specificity logistic threshold (maxSSS) was used to assess the minimum suitability of the species distribution (Liu et al., 2013a), which was visualized by QGIS 3.20.2-Odense. The predicted models were evaluated by the average AUC of ten replicate runs to determine the probability of presence locations.

We further determined the climate niches of the sampled populations by principal component analysis (PCA) using the seven environmental variables. The PCA scatterplot was visualized by the R package ggplot2 (Wickham et al., 2016). We defined the core and peripheral populations based on the probability of presence in ENM at a threshold >0.75 and the distribution patterns at the first two-axis space of the environmental PCA.

### Sampling for genetic assessment

**FIGURE 1:**
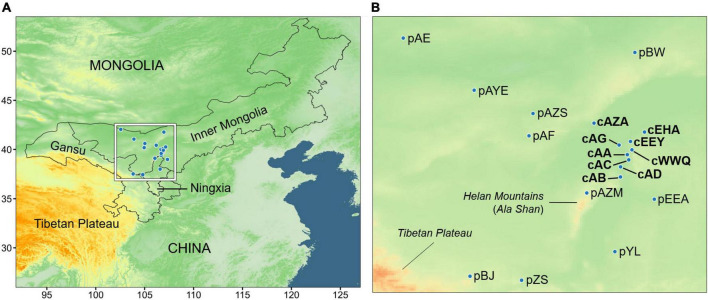
The sampling sites and the climate cohorts. **(A)** Geographic distribution of sampling sites covering three provinces (or autonomous regions) in northern China. **(B)** A close-up of the inner frame of panel **(A)**.

Based on the ENM prediction and the actual distribution, we collected a total of 217 samples from nine populations in the core region (cAZA, cAG, cAA, cAC, cAD, cAB, cEHA, cEHY, and cWWQ) and ten peripheral populations outside the suitable area (pAE, pAYE, pAZS, pAF, pBW, pEEA, pYL, pZS, pBJ, and pAZM). We prefixed “c” and “p” to represent the core and peripheral populations, respectively. The core population defined here is also the main distribution range of *A. mongolicus* in G-space, while the populations outside the core area present a more sporadic distribution. It is difficult and unrealistic to collect all sporadic peripheral populations comprehensively. Nevertheless, the sampled peripheral populations in this study have almost covered most of the entire distribution range of *A. mongolicus*, including Inner Mongolia, Ningxia, and Gansu in China (Figure 1 and Supplementary Table 2). Each population contained at least five individuals. The sample size of each population was provided in Supplementary Table 3. The fresh leaves were dried in silica gel and stored at 4°C for genomic DNA extraction.

### Molecular techniques

The genome of *A. mongolicus* is relatively large, approximately 820 Mb [2n = 18, Pang et al. (2013)], so we used the ddRAD-seq technique to obtain genome-wide genetic variations of *A. mongolicus*. Genomic DNA was extracted using the DNAquick Plant System kit (TIANGEN Biotech Co., Ltd., Beijing, China) and digested by the enzymes *RsaI* and *HaeIII*. After ligation and PCR amplification, fragment sizes of 364–414 bp were sequenced by 125 paired-end protocols on the MiSeq Illumina platform. Trimmomatic v0.38 (Bolger et al., 2014) was used to filter out adapters, poly N, and low-quality reads with default settings. The cleaned reads were mapped to the reference genome of *A. nanus*, which is the closest relative to *A. mongolicus* (Gao et al., 2018), using BWA v0.7.17 (Li and Durbin, 2009). To ensure reliability, we obtained the intersection of the SNPs from GATK v4.1.3.0 (McKenna et al., 2010) and samtools v1.10 (Li et al., 2009). For further filtration, we used vcftools v0.1.17 (Danecek et al., 2011) to retain variants with no missing data, a minor allele count above 3, a minor allele frequency above 0.01, a read depth above 3, and a minimum quality score above 100.

### Genetic diversity and population structure

#### Summary statistics

We calculated the fixation index (*F*_*ST*_), Wright’s inbreeding coefficient (*F*_*IS*_), observed (*H*o), and expected heterozygosity (*H*e), nucleotide diversity (π), and private alleles of each population using the populations program in STACKS v2.53 (Catchen et al., 2013).

#### Population genetic structure analysis

The biallelic SNPs with no missing data were used to describe the overall population genetic structure. The individual admixture coefficients were estimated using the snmf function in the R package LEA (Frichot and François, 2015). The lowest value of the cross-entropy criterion was used to evaluate the numbers of ancestral populations *K* = 1–20 and the best run among ten runs. We also employed PCA to assess the population genetic structure using the R package SNPRelate (Zheng et al., 2012).

### Genome-environment association analysis

#### Distance-based redundancy analysis

Distance-based redundancy analysis (dbRDA) was used to test the explanatory proportion of the seven climate variables for genetic composition in terms of allele frequency. We transformed the raw allele frequency data into a Bray-Curtis distance matrix (Bray and Curtis, 1957) for principal coordinate analysis (PCoA), in which the site scores on all PCoA ordination axes were used for RDA (i.e., so-called “distance-based”) to correct for differences in the variance of allele frequencies. The null model of no linear relationship between SNPs and environmental predictors was tested using *F*-statistics (Legendre et al., 2011) by the *anova.cca* function with 999 permutations. We also used the directive *by = ‘‘axis’’* to test the significance of the explanation of each axis. Candidate SNPs involved in local adaptation were then detected following Brenna R. Forester’s guidelines in “Population Genetics in R” (accessed on Feb 9, 2022^1^). Two-tailed *P* < 0.05 (standard deviation > 1.96) was set as the outlier threshold to define the candidate SNPs on each significant constraint axis. Then, the candidate adaptive SNPs were associated with each environmental variable, with the most correlated environmental variable being the potential driver of local adaptation.

#### Latent factor mixed models

Because dbRDA tests the effect of climate on overall instead of specific genetic variation, we further performed latent factor mixed model (LFMM) analysis by applying the hierarchical Bayesian method to identify the specific effect of each climate variable by controlling for residual population structure (Frichot et al., 2013). The latent factor *K*, which describes the clustering of individuals into populations adequately and the gradient of selection (Duforet-Frebourg et al., 2014), was determined by *snmf* with 100 regularizations, 200 iterations, and 10 repetitions in the LEA package. Ten replications per *K* value with 10,000 iterations and 5,000 as burn-in were performed using *lfmm* to test the associations between the seven climate variables and SNP genotypes. The *P*-value was adjusted by the *Z*-scores and genomic inflation factor (λ) to define the strength of the environmental association. The false discovery rate (FDR) was set to 0.05 to determine the environmental-associated SNPs. These SNPs were further used to conduct PCA to visualize the patterns of clustering of populations by each climate variable.

## Results

### Ecological niche modelling and climate niches

**FIGURE 2:**
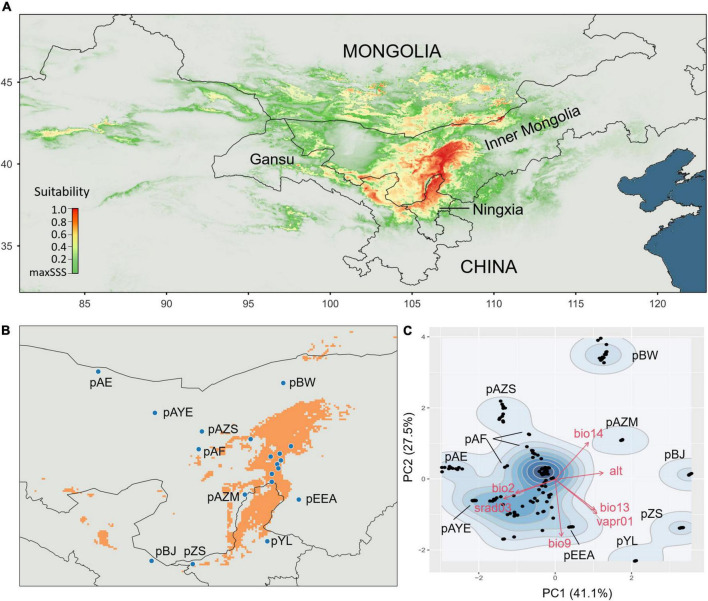
Ecological niche modelling (ENM) and climate niches. **(A)** ENM showing the potential distribution of *Ammopiptanthus mongolicus*. **(B)** The estimated suitable distribution of *A. mongolicus*. The orange colour illustrates the suitable ranges at a criterion >0.75. Peripheral populations outside the suitable ranges are explicitly labelled. **(C)** Contour plot mapped in the PCA space showing the divergence of climate niches among populations. Peripheral populations are labelled.

According to the ENM, *A. mongolicus* is mainly distributed in central and western Inner Mongolia and northern Ningxia Province (Figure 2A). The suitable growth area is roughly in the range of 37–42°N and 105–108°E, with some sporadic patches outside this area (Figure 2B). The optimal (core populations) and suboptimal growth regions (peripheral populations) were determined based on distribution modelling. The core populations clustered by similar climatic factors in the PCA, while the peripheral populations, which usually had harsh environments (Hardie and Hutchings, 2010), were mostly independently distributed in the PCA space (pBW, pAZM, pBJ, pZS, and pYL), with a few at the core edge (pAE, pAF, pAZS, and pEEA) (Figure 2C). The results of the environmental PCA were consistent with those of the ENM, supporting the determination of the core and peripheral populations.

### Sequencing and single nucleotide polymorphism calling

After sequencing and subsequent filtering steps, we obtained an average of 1,728,714 reads with an average depth of 10.1× for each sample. The cleaned reads were mapped to 996 of 1,094 contigs in the *A. nanus* genome with an average mapping rate of 84.01%. A total of 3,092,248 SNPs were identified using GATK and samtools. Following quality control, 10,705 SNPs were retained for further analysis. No samples were excluded after data quality control but only the SNPs with too-many missing were removed.

### Genetic diversity

**FIGURE 3:**
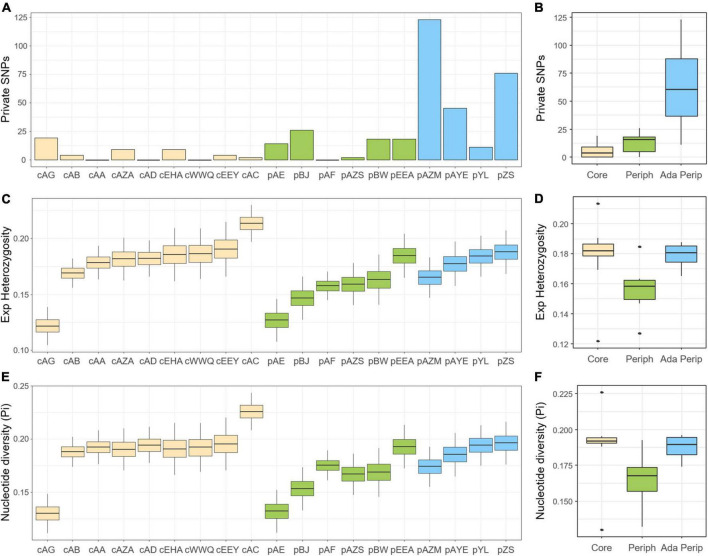
Genetic diversity of *Ammopiptanthus mongolicus* populations shown as **(A,B)** numbers of private alleles, **(C,D)** expected heterozygosity, and **(E,F)** nucleotide diversity (π). **(A,C,E)** reveal the genetic diversity distribution of all samples in each population; **(B,D,F)** reveal the average genetic diversity distribution of populations in the core, non-adaptive peripheral, and adaptive peripheral groups, denoted by yellow, green, and blue, respectively.

Based on a total of 10,705 detected SNPs, the polymorphic SNPs ranged from 2,684 (26.75%) to 7,335 SNPs (68.52%) in each population, including 0–123 private SNPs. The *H*_*O*_ estimates were between 0.163 and 0.239, the *H*_*e*_ estimates were between 0.122 and 0.213, and the nucleotide diversity (π) was between 0.130 and 0.226, indicating outcrossing or a nearly panmixia mating system (*F*_*IS*_ = -0.193 ∼ 0.009) in *A. mongolicus*. According to the sNMF, dbRDA, and LFMM, the ten peripheral populations were divided into non-adaptive (pAE, pBJ, pAF, pAZS, pBW, and pEEA) and adaptive peripheral populations (pAZM, pAYE, pYL, and pZS) (see below). The adaptive peripheral populations harboured the most private SNPs (63.75 ± 41.22 SNPs), followed by the non-adaptive peripheral populations (13.00 ± 9.22 SNPs), while the core populations had the fewest (5.22 ± 5.90 SNPs). The higher frequencies of private SNPs in the peripheral populations reflect the founder phenomenon and rapid expansion. However, compared with the core populations (*H*_*e*_ = 0.179 ± 0.023, π = 0.189 ± 0.023), genetic diversity dropped only in the non-adaptive populations (*H*_*e*_ = 0.157 ± 0.017, π = 0.165 ± 0.019) and not in the adaptive peripheral populations (*H*_*e*_ = 0.179 ± 0.009, π = 0.187 ± 0.009) (Figure 3), suggesting the evolutionary rescue potential of the adaptative peripheral populations. Details of genetic diversity indices are listed in Supplementary Table 3.

### Population structure

**FIGURE 4:**
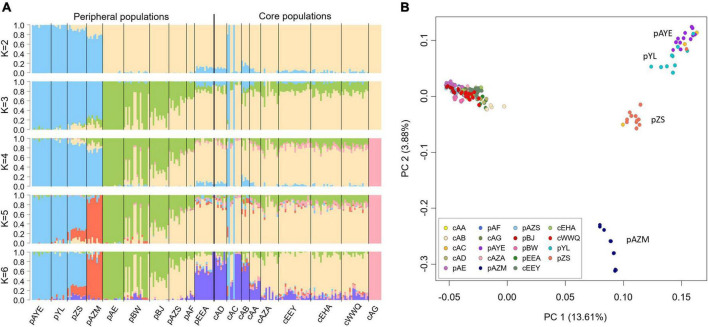
Population genetic structure of *Ammopiptanthus mongolicus* inferred by **(A)** sNMF and **(B)** PCA with all SNPs. The optimal *K* of sNMF is 2, and *K* = 3–6 are shown to reveal detailed structure patterns.

The best clustering number of sNMF was *K* = 2, which separated the peripheral populations pAYE, pYL, pZS, and pAZM from the other populations (Figure 4A). Three samples of cAC had genetic components identical to these peripheral populations, suggesting that they were dispersers. When *K* > 3, a minor genetic component of the core population (i.e., the green part of Figure 4A) expanded in the peripheral populations, suggesting a founder direction from the core to the periphery. When *K* = 4, cAG displayed a unique genetic component. In finer clusterings (*K* = 5 or 6), pAZM was separated as an independent group, and pZS was admixed between pAZM and pAYE+pYL (Figure 4A), consistent with the PCA results: pAYE was closest to pYL, pAZM was independently separated, and pZS was in the middle (Figure 4B). Although the proportions of genetic components differed between the other populations at *K* > 3 in sNMF and cAG was completely segregated at *K* = 4, they were all grouped together in the PCA (Figure 4B).

**FIGURE 5:**
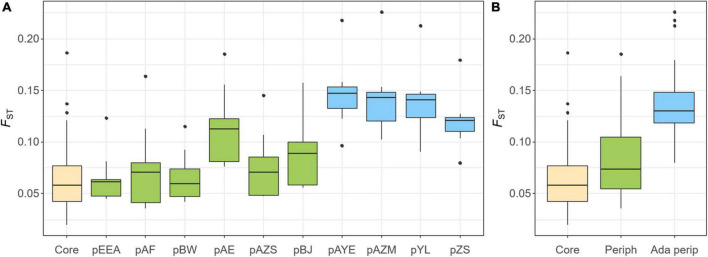
Population genetic differentiation (*F*_*ST*_) within the core populations (yellow), between the core and non-adaptive peripheral populations (green), and between the core and adaptive peripheral populations (blue). *F*_*ST*_ is displayed at **(A)** the population level and **(B)** the group level of core, non-adaptive peripheral, and adaptive peripheral populations.

The pairwise *F*_*ST*_ was estimated to compare the extent of gene flow (i.e., the reverse of genetic differentiation) within the core populations and between the core and peripheral populations (Figure 5). *F*_*ST*_ was small within the core populations, with a value of 0.066 ± 0.036, and was slightly higher between the core and non-adaptive peripheral populations (*F*_*ST*_ = 0.082 ± 0.035, ranging from 0.065 to 0.115, Figure 5). However, genetic differentiation increased significantly between the core and adaptive peripheral populations (*F*_*ST*_ = 0.136 ± 0.033, ranging from 0.120 to 0.146, Figure 5). These findings are congruent with the sNMF and PCA results.

### Distance-based redundancy analysis

**FIGURE 6:**
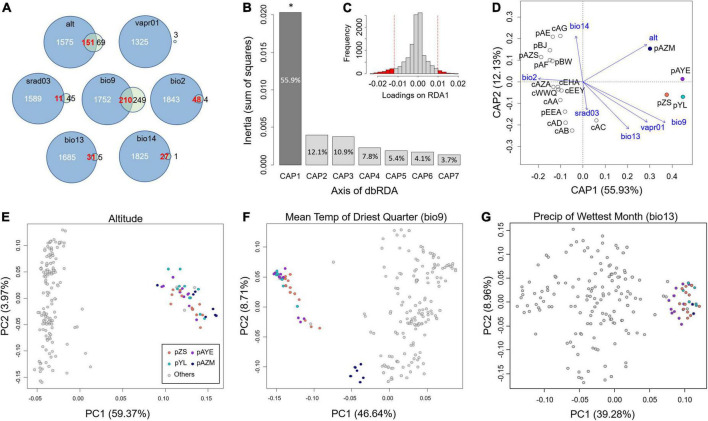
Determination of the driver of local adaptation. **(A)** The intersection of putative adaptive SNPs inferred by LFMM (blue circles) and dbRDA (green circles). The values denote the estimated number of adaptive SNPs. **(B)** The explanatory proportion of each axis of the dbRDA, where only the 1st axis (CAP1) has a significant effect. **(C)** The distribution of SNP loadings on the 1st axis of the dbRDA. Red vertical lines denote the threshold of 95% confidence intervals to determine outliers (i.e., putative adaptive SNPs). **(D)** Scatter plot of the seven climatic parameters in the space of the first two axes of the dbRDA. **(E–G)** PCA scatterplots of the adaptive SNPs associated with environmental factors, including **(E)** altitude, **(F)** bio9, and **(G)** bio13, inferred by the intersection of the dbRDA and LFMM. The four coloured populations are the inferred adaptive peripheral populations.

The full model of distance-based redundancy analysis (dbRDA) that significantly rejected the null model (*F* = 1.967, *P* = 0.018) revealed that environmental factors explained 55.58% of the overall genetic variation. Among all transformed axes of the dbRDA, only the 1st axis significantly affected genetic variation (55.93% variation of the constraint variables, *F* = 7.699, *P* = 0.038, Figure 6A). We therefore searched for potential adaptive SNPs on the 1st axis, and a total of 854 outliers were detected (Figure 6B), of which 459 SNPs were associated with the mean temperature of the driest quarter (bio9), 220 SNPs were altitude associated, and the remaining 175 SNPs were associated with the other five climate factors.

The dbRDA scatter plot showed that four populations, pAZM, pAYE, pZS, and pYL, were separated from the others by 1st axis (Figure 6D). In the space of the first two axes, these four populations were closest to the original vectors of alt and bio9 (Figure 6C), consistent with an effect of the environmental factors on the most adaptive SNPs (Figure 6A). The contour plots showed that the genetic variation of the four populations was marginally (*P* = 0.090) associated with higher elevation, with an explanatory proportion of 7.8% (Supplementary Figure 2); however, the genetic variation was scattered across a gradient of bio9 (4.3%, *P* = 0.312) and the other environmental factors with non-significant and relatively low explanations (Supplementary Figure 2).

### Latent factor mixed model

Latent factor mixed model identified more environment-associated SNPs than dbRDA, with a range of 1,325–1,962 for each climate factor and altitude. We took the intersection of the environment-associated SNPs estimated by dbRDA and LFMM as the adaptive SNPs. There was no intersection of SNPs for vapr01, but the other six environmental factors had 210 (bio9), 151 (alt), 48 (bio2), 31 (bio13), 27 (bio14), and 11 (srad03) associated SNPs (Figure 6A).

We then performed PCA on these environment-associated adaptive SNPs to examine their groupings. The results showed that altitude and bio13 clearly separated pZS, pAYE, pYL, and pAZM from the other populations (Figures 6E,G and Supplementary Figure 3). These four populations were also separated from the other populations by adaptive SNPs of bio9, with further separation of pAZM alone (Figure 6F). These four populations were separated from the others by srad03 in PC1 but with weak groupings (Supplementary Figure 3). Bio14 and bio2 divided all the populations into three groups (Supplementary Figure 3), and pZS, pAYE, pYL, and pAZM were congregated within one group by bio14 but scattered into three groups by bio2. In short, we identified altitude, bio13, and bio9 as the leading environmental variables differentiating pZS, pAYE, pYL, and pAZM from the other populations (Figures 6E–G).

## Discussion

Unlike the apparent population genetic clustering inferred by dominant markers (Ge et al., 2005; Jiang et al., 2019), the genome-wide SNPs showed relatively homogeneous genetic variation among the populations, reflecting the low genetic diversity of *A. mongolicus* compared with other desert plants [see Jiang et al. (2019)]. Jiang et al. (2019) suggested that the local climate, especially summer precipitation, restricts the gene flow of *A. mongolicus*, thus explaining the apparent population genetic differentiation. The genome-wide SNPs also confirmed the IBE inferred by Jiang et al. (2019) (Supplementary Table 4). This speculation represented a modification of Ge et al. (2005) hypothesis of IBD. However, previous work was limited to genetic markers and thus mainly tested the correlations between genetic differentiation and environmental or geographic distances (i.e., IBE or IBD) while ignoring the specific effect of local climate on the filtering of local genes. This study focused on local climate effects to identify locally adapted populations, thus filling a gap in understanding the impact of environment-leading selection on the only broad-leaved evergreen shrub in the Asian temperate desert.

### Determining peripheral populations by differentiating local climates from the core

The core-periphery hypothesis predicts that the core region best satisfies the niche requirements of the species, while peripheral populations increasingly experience unfavourable ecological conditions (Brown, 1984). Therefore, peripheral populations are determined by environmental differences instead of geographic distance from the core (Duncan et al., 2015). In the case of *A. mongolicus*, the environmental peripheral populations are not necessarily at the geographical margins. For example, pAZM, which is close to the geographic core, was defined as a peripheral population by high loadings of altitude and precipitation in the driest month (bio14) (Figure 2c). This core-periphery definition by environment also coincides with the conclusion that environmental differences are the limiting factor in the dispersal of *A. mongolicus* (Jiang et al., 2019). Under PNC, a species’ range is constrained by niche limits and dispersal ability even across continuous environmental gradients (Hargreaves et al., 2014), which is particularly appropriate for describing the core distribution of *A. mongolicus*. If the core area is optimal for growth and reproduction, the core populations of *A. mongolicus* should be the legacy of adaptation to cold and arid environments. By contrast, genetic surfing, which increases the frequency of rare alleles toward the margins, combined with spatial sorting of selection at the margins, could break the niche limits to allow adaptation to heterogeneous environments, facilitating range expansion (Angert et al., 2020).

Peripheral populations defined according to the E-space concept share the same characteristics as G-space edge populations: low genetic diversity, high frequencies of rare and novel alleles, and low genetic supplementation (high *F*_*ST*_) from the core to peripheral populations. A notable exception is the core population cAG defined by E-space, which possesses genetic characteristics like a peripheral population. Almost identical genotypes with high frequencies of private alleles in cAG samples suggest a newly established population. This new population may be expanded from a few founders or tillered from the adventitious roots of broken branches. The four peripheral populations (pZS, pAYE, pYL, and pAZM) form distinct clusters with respect to altitude- and climate-associated SNPs as a result of spatial selection and sorting, whereas the other peripheral populations have fewer private SNPs with low genetic diversity, indicating that adaptation did not occur.

### Surfing of new mutations from standing variation shifts the adaptive optima in adaptive peripheral populations

Founder and bottleneck effects lowered the genetic diversity of *A. mongolicus* in peripheral populations. Coupled with the selection pressure caused by the core-periphery environmental differences, peripheral populations may face higher extirpation risk (Fady et al., 2016). Genetic replenishment may not mitigate this extirpation risk; instead, such a genetic rescue may inhibit peripheral populations from evolving toward local ecological optima (García-Ramos and Kirkpatrick, 1997), consistent with the genetic swamping hypothesis (Haldane, 1956). However, if the selection is stronger than swamping, peripheral populations tend to diverge from the species’ core (Hardie and Hutchings, 2010). In other words, in this case, limited gene flow suppressed by local environmental differences (i.e., IBE) (Jiang et al., 2019) prevented genetic swamping and led to shifts in adaptive optima in the four adaptive peripheral populations with high genetic diversity (Stetter et al., 2018).

Populations subjected to environmental margins, low gene flow, and high genetic diversity have higher adaptive potential, as exemplified by pZS, pAYE, pYL, and pAZM. Abundant private SNPs were detected in these adaptive peripheral populations, consistent with the genetic surfing hypothesis, which posits that new mutations are quickly fixed in the wavefront populations (Excoffier and Ray, 2008). The genetic signature of surfing is characterized by rare core genetic components becoming prevalent in the distal periphery with a strong spatial-genetic structure (Figure 4; Graciá et al., 2013). However, the similar inbreeding coefficient (*F*_*IS*_) between the core and the adaptive peripheral populations indicates that mating systems are unaltered by distribution (Supplementary Figure 4). Meanwhile, the higher observed heterozygosity (*H*_*O*_) in peripheral populations not only suggests the newly derived mutations from standing variations but also implies the environmental tolerance for alloploidy, i.e., relaxation of selective constraints (Supplementary Figure 4). These population genetic characteristics assist the adaptive optima shift in peripheral populations (Stetter et al., 2018).

### Elevational adaptation of mountainous peripheral populations

The desert environment is relatively stable compared with other landscapes. Thus, range expansion should be less hindered in desert environments. However, low spatial-environmental variation (i.e., ΔE) may constrain adaptative flexibility (i.e., adaptability) in desert organisms because of the reduction in the G × E effect (Brooker et al., 2022). Altitude differences increase environmental heterogeneity in deserts, enhancing the edge effect in desert plants. Subsequent divergent selection maintains the genetic differences between populations and magnifies the G × E impact. In the Helan Mountains, where the population pAZM is located, altitude-related climate dissimilarities reduce effective gene flow from the nearby core populations, leading to a pattern of isolation-by-adaptation (Nosil et al., 2008). A previous study also showed that the population (Beisi, 38.98°N, 105.87°E, 1726 m a.s.l.) near pAZM has distinct ITS and cpDNA haplotypes compared with other populations of *A. mongolicus* (Su et al., 2016). Such genetic differentiation of low-substitution-rate markers implies that the altitude-related divergence is longstanding.

Elevation is a topographic factor involving multiple climate factors (Korner, 2007). The climatic factors highly correlated with altitude (*R*^2^ > 0.6 and *P* < 0.0001) were bio5 (maximum temperature of warmest month, *r* = -0.8589), bio7 (temperature annual range, *r* = -0.8401), bio10 (mean temperature of the warmest quarter, *r* = -0.8602), and summer solar radiation (srad6, *r* = -0.8136; srad7, *r* = -0.8401; srad8, *r* = -0.8225). Thus, altitudinal adaptation appears to be related to summer temperature and sunshine. In deserts, summer is the rainy season, which is the regeneration and growing season of this desert shrub. The negative correlations (i.e., the decreasing values of these summer climatic factors along altitude) suggest that low light and low temperature in the mountains adversely affect the growth of *A. mongolicus*. The distinct grouping of altitude-related SNPs demonstrates adaptive changes in these four adaptive peripheral populations in high-altitude environments, despite the unclear functions of these anonymous SNPs.

### Pleiotropic adaptative divergence to cold and drought stresses

Winter temperatures place severe stress on temperate shrubs, which in turn accelerates the evolution of cold-resistant mechanisms (Novák et al., 2021). Despite being anonymous markers, SNPs were selected the most by winter temperature (i.e., bio9, see Supplementary Figure 1) in either the intersection (210 SNPs) or union of LFMM and dbRDA (1,752 + 210 + 249 = 2,211 SNPs, see Figure 6A), reflecting a marked adaptive cue under cold stress. As a broad-leaved evergreen shrub, *A. mongolicus* must rely on physiological adaptation to temperature fluctuations because cold damage cannot be prevented via physical defoliation (Pang et al., 2013; Wu et al., 2014). However, the adaptive peripheral populations may not exhibit cold stress tolerance because the relatively high winter temperature frees them (except for pAZM) from the selective pressure of cold stress (Figure 6F and Supplementary Figure 1A). Among these four populations, pAZM was closer to the other core populations and non-adaptive peripheral populations in the PCA, indicating that the mountainous pAZM population was still adapted to the cold winter.

In addition to cold stress, summer precipitation (i.e., bio13) diverged the four adaptive peripheral populations from the other populations, revealing adaptive divergence. *A. mongolicus* is a xeric plant that grows in rocky, gravelly, sandy soils of rocky dunes with a soil depth of less than 30 cm (Liu, 1998). Moisture is one of the main ecological factors limiting the distribution of *A. mongolicus* (Liu et al., 2017). *A. mongolicus* flowers from April to May and fruits from May to June. Summer rainfall limits its regeneration (Liu et al., 2013b; Jiang et al., 2019). Three of the four adaptive peripheral populations (pAZM, pYL, and pZS) inhabit relatively humid environments in summer, whereas the core and non-adaptive peripheral populations are located in areas with drier summer and relatively unfavourable for regeneration (Supplementary Figure 1). The adaptively divergent SNPs identified by LFMM and dbRDA may be associated with drought tolerance in these populations. Since the diaspore dispersal and entomophilous pollination of *A. mongolicus* require dry conditions (Jiang et al., 2019; Yang et al., 2021), a wetter summer allows these adaptive peripheral populations to germinate rapidly but hinders gene flow (Figure 5). This ecological mechanism may accelerate the process of adaptive divergence between core and peripheral populations.

Accumulating evidence indicates that physiological regulation and adaptation under cold and drought stresses are controlled by the same genes in *A. mongolicus*. Under long-term aridity and extremely cold conditions, 1,594 cold and drought unigene sets (namely the AmCDUnigene set) were identified (Liu et al., 2013b). Similarly, 971 DEGs co-regulated by both cold and drought stresses were identified, with enrichment of flavonoid biosynthesis genes and membrane protein genes, among others (Wu et al., 2014). The responses of *A. mongolicus* to cold and drought stresses are co-regulated by *AmGORK*, which responds to stomatal closure (Li et al., 2016); dehydrin genes (*AmDHN132*, *AmDHN154*, and *AmDHN200*), which respond to dehydration stress (Cui et al., 2020); and dehydration-responsive element-binding (DREB) transcription factors (TFs) (*AmDREB1F*, *AmDREB2C*, and *AmDREB3*) (Yin et al., 2018; Ren et al., 2019; Tang et al., 2021) and NAC TFs (e.g., *AmNAC11*) (Pang et al., 2019). These studies indicate that *A. mongolicus* may use the same or similar physiological strategies to respond to different seasonal heterogeneous environmental stresses via pleiotropy (van Boheemen and Hodgins, 2020; Yuan and Stinchcombe, 2020). However, these studies emphasized differential gene expression without examining genetic variation between populations. Despite the use of anonymous SNPs, our study points to an association between environment and genetic variation, complementing the lack of field observations in previous studies. Since both drought and cold are characteristic of temperate deserts, adapting the same genes to such environmental adversities may be the most parsimonious evolutionary strategy, which explains the similar clustering patterns of bio9 and bio13 in the PCA.

The above cold and drought adaptation mechanisms suggest that the selected populations are not the four so-called adaptive peripheral populations but the others (core and non-adaptive peripheral populations). Du et al. (2021) predicted range expansion and habitat shift with increasing temperature, suggesting that a warmer climate is more suitable for *A. mongolicus*. Thus, relaxation of selective constraints more readily explains the genetic divergence of these four adaptive peripheral populations, which is also supported by the increase in new mutations (private SNPs). By contrast, the core populations underwent long-term adaptation ca. 4.5 Mya to the harsh desert environment after the rapid uplift of the Qinghai-Tibetan Plateau (Shi et al., 2017). That is, adaptive divergence in *A. mongolicus* is a consequence of the relaxation in peripheral populations of selective constraints from the adaptive legacy of the core populations.

### Concluding remarks: Pleiotropic selection may facilitate the parallel adaptation of peripheral populations

In relatively homogeneous desert environments, it is difficult to track adaptations to heterogeneous environments in the context of a long-standing adaptive legacy. This study demonstrates that peripheral populations have a greater chance of escaping the selective constraints of harsh environments by acquiring and retaining new mutations. The (adaptive) optimal shift at high altitude and the relaxation of cold and drought pressures were associated with convergence of the genetic characteristics of allopatric peripheral populations according to the phenomenon of parallel adaptation. Clustering in populations exposed to similar (but not identical) multiple environmental stresses suggests the importance of pleiotropic selection in adaptation to dramatic annual climate fluctuations in temperate deserts. This study provides not only a more accurate species distribution and spatial-genetic structure of *A. mongolicus* but also evidence that adaptive divergence may occur through local adaptation and through escape from long-term adaptive constraints via pleiotropy.

## Data availability statement

The datasets presented in this study can be found in online repositories. The names of the repository/repositories and accession number(s) can be found below: https://data.mendeley.com/, DOI: 10.17632/9g8fd4t2nj.2.

## Author contributions

R-HG and P-CL conceived and designed the research. L-DP provided and checked all occurrence data of the study species. Y-ZY, L-DP, and R-HG collected the samples. Y-ZY and M-XL conducted the experiments. M-XL, J-TC, and P-CL analyzed the data. P-CL wrote the draft with the input of M-XL and J-TC. All authors read and agreed to the published version of the manuscript.

## Footnotes

1
https://popgen.nescent.org/

## Supplementary material

Supplementary Table 1 Occurrence data of *Ammopiptanthus mongolicus* for ENM (csv file).

Supplementary Table 2 Detailed sampling information (csv file).

Supplementary Table 3 Sample size and genetic diversity indices of each population (csv file).

Supplementary Table 4 Maximum-likelihood population-effects (MLPE) mixed models for testing the isolation-by-environment (IBE) and isolation-by-distance (IBD) models (word file).

Supplementary Method R codes and input files used in this study (zip file).
