# Supplementary material for: Parallel adaptation prompted core-periphery divergence of Ammopiptanthus mongolicus
Source: Front Plant Sci. 2022 Aug 24;13:956374. doi: 10.3389/fpls.2022.956374 (PMC9449729; doi:10.3389/fpls.2022.956374)
Supplement: Supplementary file 1 [file Data_Sheet_5.docx]

Supplementary Figures


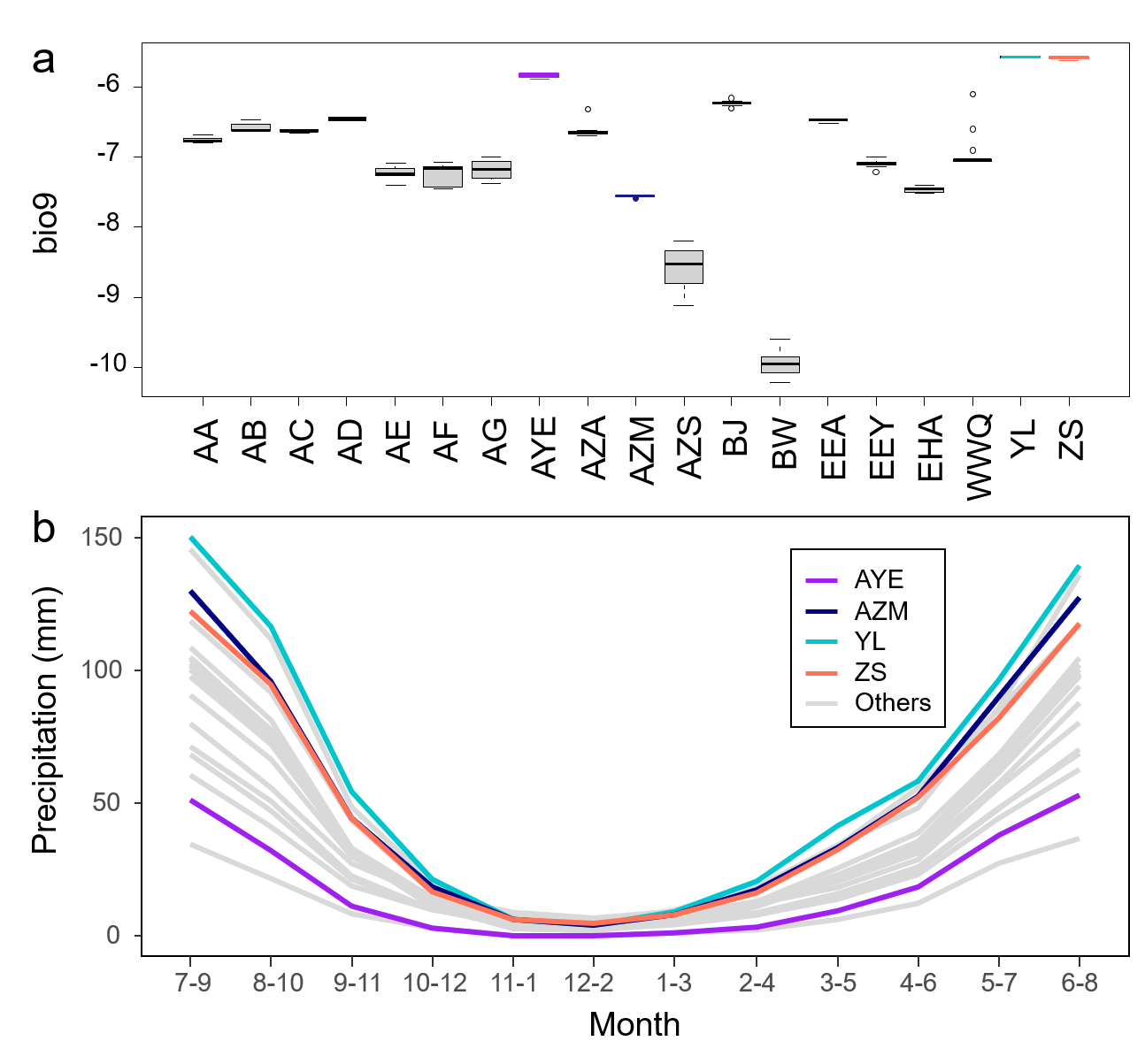


**Fig. S1** Mean temperature of the driest quarter (bio9) in each population. (a) Box plot showing the regional differences in bio9. (b) Precipitation of the 12 quarters, showing the driest season (quarter).


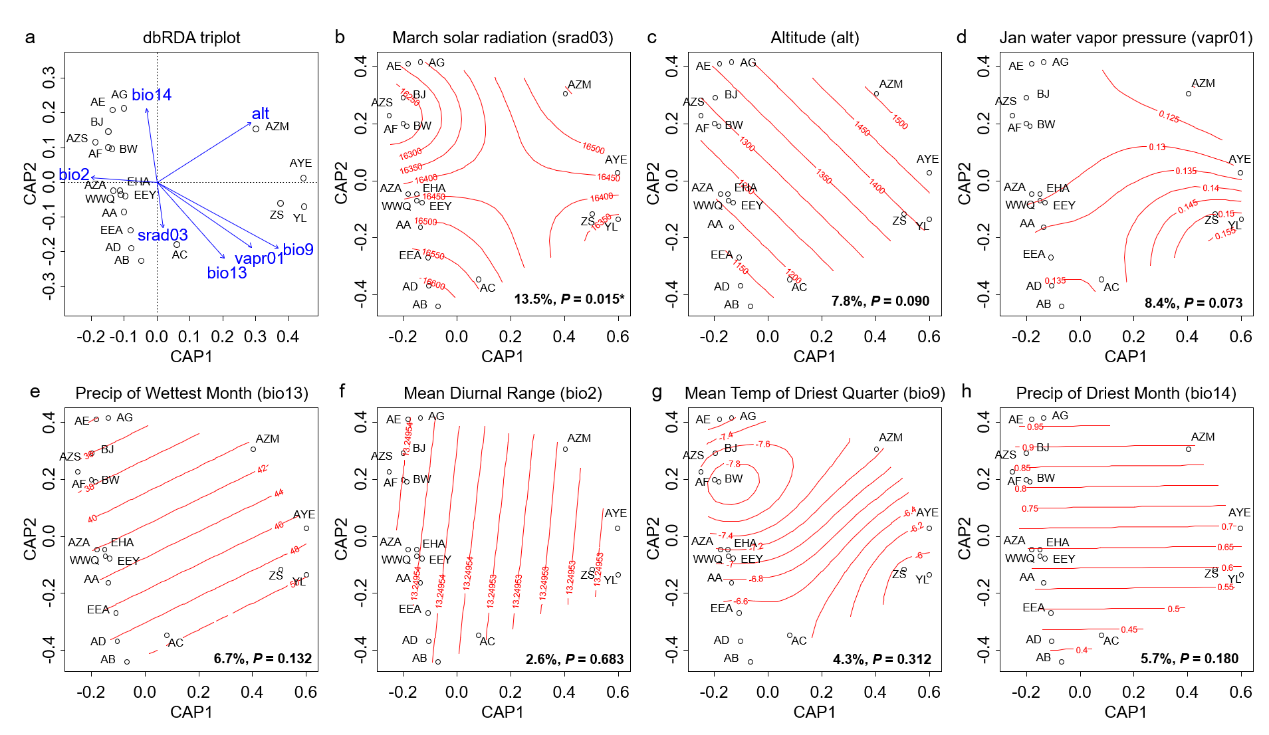


**Fig. S2** Scatterplot and contour plots of the dbRDA. (a) Scatterplot of the dbRDA; (b)~(h) Contour plots of (b) srad03, (c) altitude, (d) vapr01, (e) bio13, (f) bio2, (g) bio9, and (h) bio14.


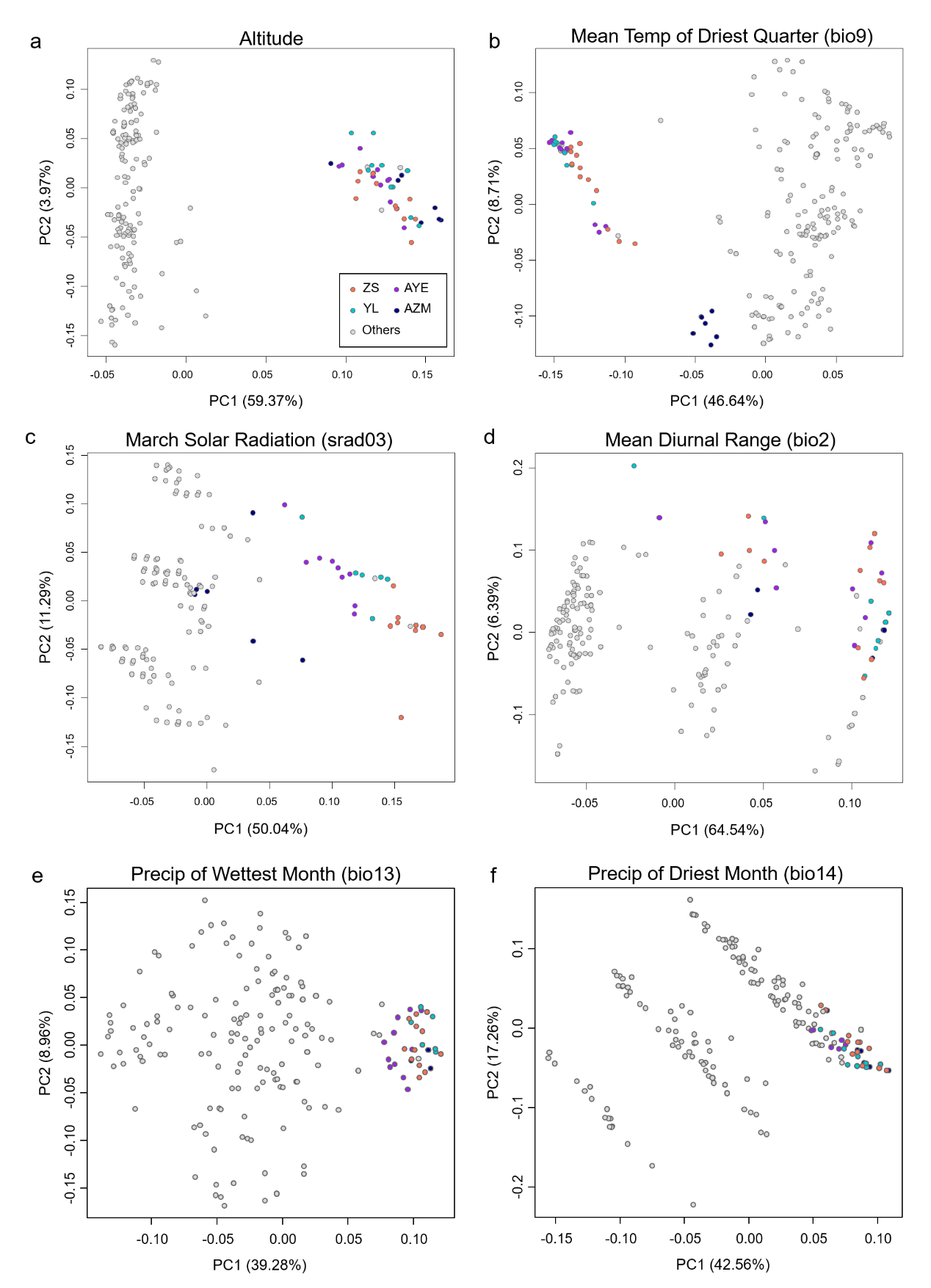


**Fig. S3** PCA of SNPs associated with environmental factors: (a) altitude, (b) bio9, (c) srad03, (d) bio2, (e) bio13, and (f) bio14. No adaptive SNPs associated with vapr01 were detected. The four colored populations are the inferred adaptive peripheral populations.


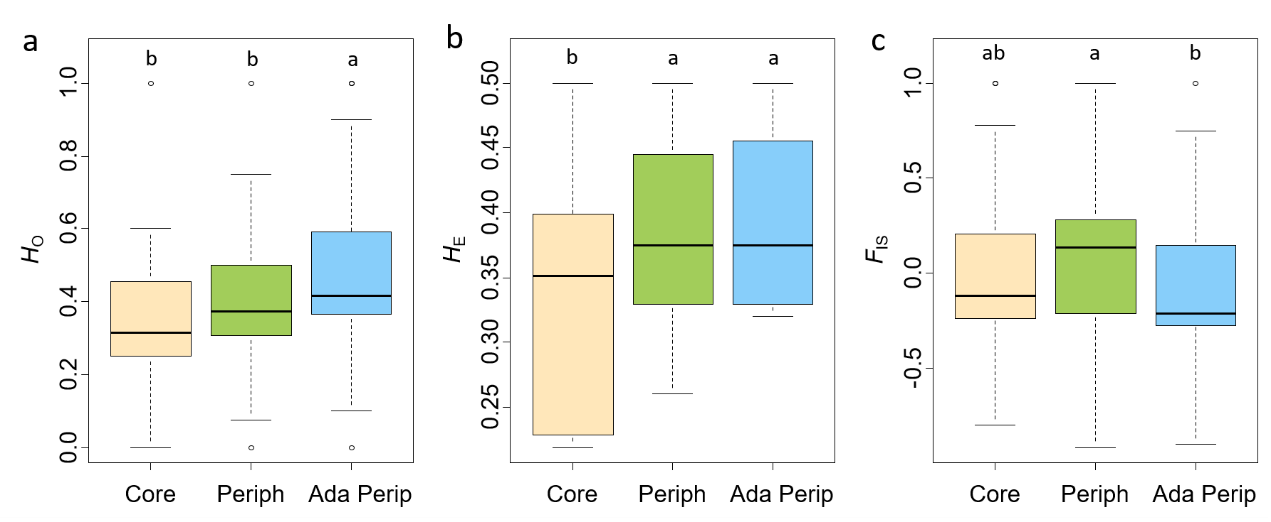


**Fig. S4** Heterozygosity and *F*-statistic (inbreeding coefficient) of the private SNPs. (a) Observed heterozygosity; (b) expected heterozygosity; (c) *F*-statistic (*F*_IS_). These three genetic diversity indices were significantly different among the groups according to one-way ANOVA (*P* = 1.27×10^-5^, 5.77×10^-6^, and 2.38×10^-3^ in *H*_O_, *H*_E_, and *F*_IS_, respectively). The *post-hoc* HSD results are shown above the boxplots in lowercase letters. This comparison excludes population AG, which is probably a clonal colony (i.e., genet) because all samples had nearly identical genotypes.
