# Supplementary material for: Parallel adaptation prompted core-periphery divergence of Ammopiptanthus mongolicus
Source: Front Plant Sci. 2022 Aug 24;13:956374. doi: 10.3389/fpls.2022.956374 (PMC9449729; doi:10.3389/fpls.2022.956374)
Supplement: Supplementary Table 4 — Maximum-likelihood population-effects (MLPE) mixed models for testing the isolation-by-environment (IBE) and isolation-by-distance (IBD) models (word file). [file Data_Sheet_6.docx]

**Maximum‐likelihood population‐effects (MLPE) mixed models for testing the IBE and IBD**

To confirm the isolation-by-environment (IBE) hypothesis, the maximum‐likelihood population‐effects (MLPE) mixed model was performed to compare the IBE with the isolation-by-distance (IBD) model. The Euclidean geographic distance was calculated for constructing a geographic-distance matrix, and a Brey-Curtis environmental distance matrix was constructed using all 55 environmental variables used in this study. The variable altitude was excluded in IBE because it should be a topological instead of an environmental factor. Genetic distance was calculated using the formula Fst/(1-Fst). The result showed lower AIC and BIC values and a higher log-likelihood (logLik) in IBE than in IBD (ΔAIC = -6.5257, ΔBIC = -6.5258, ΔlogLik = 3.263, Table S4), indicating a better-fit of IBE model than the IBD in explaining the population genetic differentiation. The MLPE result confirms the previous conclusion of IBE by Jiang et al. (2019).

**Table S4** Maximum‐likelihood population‐effects (MLPE) mixed models for testing the isolation-by-environment (IBE) and isolation-by-distance (IBD) models

|  | AIC | BIC | logLik | deviance | df.resid |
| --- | --- | --- | --- | --- | --- |
| IBE | -611.220 | -598.654 | 309.610 | -619.220 | 167 |
| IBD | -604.695 | -592.128 | 306.347 | -612.695 | 167 |
